# Generative adversarial network based data augmentation for CNN based detection of Covid-19

**DOI:** 10.1038/s41598-022-23692-x

**Published:** 2022-11-10

**Authors:** Rutwik Gulakala, Bernd Markert, Marcus Stoffel

**Affiliations:** Institute of General Mechanics, RWTH Aachen University, Aachen, Germany

**Keywords:** Biomedical engineering, Image processing, Machine learning

## Abstract

Covid-19 has been a global concern since 2019, crippling the world economy and health. Biological diagnostic tools have since been developed to identify the virus from bodily fluids and since the virus causes pneumonia, which results in lung inflammation, the presence of the virus can also be detected using medical imaging by expert radiologists. The success of each diagnostic method is measured by the hit rate for identifying Covid infections. However, the access for people to each diagnosis tool can be limited, depending on the geographic region and, since Covid treatment denotes a race against time, the diagnosis duration plays an important role. Hospitals with X-ray opportunities are widely distributed all over the world, so a method investigating lung X-ray images for possible Covid-19 infections would offer itself. Promising results have been achieved in the literature in automatically detecting the virus using medical images like CT scans and X-rays using supervised artificial neural network algorithms. One of the major drawbacks of supervised learning models is that they require enormous amounts of data to train, and generalize on new data. In this study, we develop a Swish activated, Instance and Batch normalized Residual U-Net GAN with dense blocks and skip connections to create synthetic and augmented data for training. The proposed GAN architecture, due to the presence of instance normalization and swish activation, can deal with the randomness of luminosity, that arises due to different sources of X-ray images better than the classical architecture and generate realistic-looking synthetic data. Also, the radiology equipment is not generally computationally efficient. They cannot efficiently run state-of-the-art deep neural networks such as DenseNet and ResNet effectively. Hence, we propose a novel CNN architecture that is 40% lighter and more accurate than state-of-the-art CNN networks. Multi-class classification of the three classes of chest X-rays (CXR), ie Covid-19, healthy and Pneumonia, is performed using the proposed model which had an extremely high test accuracy of 99.2% which has not been achieved in any previous studies in the literature. Based on the mentioned criteria for developing Corona infection diagnosis, in the present study, an Artificial Intelligence based method is proposed, resulting in a rapid diagnostic tool for Covid infections based on generative adversarial and convolutional neural networks. The benefit will be a high accuracy of lung infection identification with 99% accuracy. This could lead to a support tool that helps in rapid diagnosis, and an accessible Covid identification method using CXR images.

## Introduction

Infections caused by the Covid-19 strain were upgraded to pandemic status in 2020 after it was declared a worldwide emergency^1^. As of 16 September 2022, there are over 600,328,548 confirmed cases of Coivd-19 including 6,501,469 deaths as reported by WHO and John Hopkins university Covid-19 dashboards^2,3^. Subsequently, techniques for diagnosing coronavirus infections were developed, including the polymerase chain reaction (PCR) test^4^ and the reverse transcription polymerase (RT-PCR) chain reaction test^5,6^. As an alternative to traditional diagnostic techniques, a lung screening procedure gained popularity in the literature since it could quickly spot signs of Covid-19 infections using X-ray image recognition^5,7,8^. Applications for convolutional neural networks (CNN) are numerous and varied. They are mostly utilized in image analysis^9,10^, for eg. mechanobiological applications^11^, but they are also used in complex regression problems^12–14^, for eg. function approximation in experimental investigations^15,16^. CNNs can therefore discriminate between lung scans that are infected and those that are not. However, these networks require a significant amount of training data, or X-ray images, in order to achieve high accuracy and compete with biological evaluations for this sense. The neural network must extract significant information from lung X-rays in order to find disease markers. Because of this, CNNs and autoencoders were suggested in the literature^17^. In^18^, various CNN topologies were also suggested as a way to increase classification accuracy. For the same reason, researchers looked into different architectures of AI, including Scalable Vector Machines (SVM)^17,19^. In^20^, a CNN was used to distinguish between infected lungs, non-infected lungs, and common pneumonia. Artificial neural networks that have been further developed and are capable of detecting lung illnesses have also been used, for example, to diagnose tuberculosis^21^. The quantity of data available for training the CNN determines how well neural network classification techniques perform. Due to the scarcity of Covid-19 chest X-ray images, data augmentation techniques were used to boost the training dataset. Generative learning based methods were also proposed in the literature to augment training data and generate synthetic data thereby boosting the training dataset. CovidGAN is one such architecture, proposed in^22^ to improve the accuracy of neural network classifications of lung scans, boosting the accuracy up to 95%.

Most of the models proposed in the literature perform binary classification of Covid-19 positive and negative classes and the very few multi-class classification models that are available in the literature for Covid studies, don’t have very high test accuracy. Many studies in the literature have used a standalone GAN to perform the classification of chest X-Rays instead of using CNNs. To perform multiclass classification, some studies have coupled two different GAN architectures to achieve this. Not only is this computationally extensive to train and deploy, but also doesn’t achieve very high accuracy. Therefore, in this study, we propose a decoupled approach to maximize accuracy and make the models deployable on radiology equipment. Since the X-ray images used in this study are collected from different sources, and the public data banks have images compiled from different hospitals and they are not captured using the same kind of machines with the same settings. This results in randomness in the luminosity of the X-ray images and this can cause problems during training. Also, the radiology equipment cannot run state-of-the-art CNN architectures like ResNet and DenseNet effectively since they have millions of parameters. To overcome these limitations, a novel CNN architecture is proposed to perform multi-class classification of Covid-19, healthy and pneumonia classes, that is 40% lighter and can deal with randomness in luminosity better, leading to better accuracy than state-of-the-art models. With a combined GAN-based data augmentation and a CNN-based classification by introducing a Residual U-Net GAN for generating synthetic chest X-ray pictures, followed by CNN training, an accuracy of 99% has been achieved by the proposed method.

## Related work

In recent years, several studies have been proposed, using GANs and CNNs for Covid-19 detection tasks. A supervised deep learning model based on CNN called COVID-NET has been proposed in^23^ leading to 93.3% test accuracy, on a test set of 100 samples of normal, pneumonia, and Covid-19 chest X-ray (CXR) images from the COVIDx dataset^24^. All other images were used for training the model. A combined approach involving YOLO algorithm to detect and isolate the chest portion of X-ray and a modified VGG19 CNN architecture has been proposed in^25^ to classify between Covid-19, pneumonia and healthy CXR images where the authors have achieved a very high accuracy of 99.84% for binary classification and 97.16% for multi-class classification. In^26^, the binary and multiclass classification of CXR images using the Darknet neural network model was investigated. They achieved a reported binary classification accuracy of $98.08\%$ and multi-class classification with accuracy of $87\%$ on 25 Covid-19, 100 normal, and 100 pneumonia images. Multiple architectures such as VGG19, DenseNet121, and InceptionV3 are investigated in^27^ and the mentioned architectures are tested on a small set of 25 Covid-19 positive and 25 Covid-19 negative images, with reported accuracies of between 50% (InceptionV3) to 90% (VGG19 and DenseNet201) for each investigated architecture. A novel framework was developed in^28^ for rapid diagnosis of Covid-19 on CT scans using CNNs based on a Naive Bayes classifier to classify Covid-19 and healthy cases, where the authors used multiple strategies to extract features and a genetic algorithm to select appropriate features from the inputs with 92.6% accuracy. A channel boosted CNN was proposed in^29^ which has shown an excellent detection rate of 97%. The major drawback in all these studies is limited availability of data and the size of the test set used to evaluate the model. Since there is very less open source data available for CXR images affected with Covid-19, all the available studies could not use more than 50 test images for testing the model.

One of the hurdles in dealing with medical images is data labeling. It is extremely tedious, laborious, expensive, and accurate annotation of images demand expert knowledge of doctors. Many methods have been proposed in the literature to augment available data to increase the size of the training set and thus achieve better accuracy. In^30^, the authors have utilized multiple image augmentation techniques such as random flipping, random jitter and random cropping to augment the input pipeline. Semi-automated and automated classification algorithms have been proposed in^31,32^ to overcome this issue to classify unlabeled data. In addition to classical augmentation methods, Deep learning based augmentation techniques have been gaining a lot of momentum in recent years, especially Generative modelling based methods such as GANs^33^. In^34^, a GAN based data augmentation has been proposed for improving cancer classification on gene expression data, where the authors achieved an 18.8% improvement in classification accuracy as a result of the proposed augmentation method. Algorithms like Auxillary classifier GAN (ACGAN), Deep Convolutional GAN (DCGAN), CycleGAN, pix2pix and progressively growing GANs have proven to be efficient GAN based image augmentation techniques^30,35–38^. Many studies have been published using these GAN based augmentation techniques in CXR detection. CovidGAN was proposed in^22^, which is based on an Auxilary Classifier GAN^35^, being able to generate synthetic data and thereby improving the accuracy of classification. Using this method, improved CNNs with accuracy from 85 to 95% were reported. GAN based data augmentation has been performed in^39^ for CXR classification which resulted in a 3% increase in the accuracy. RANDGAN has been proposed in^40^ to classify images of unknown classes from known classes such as normal and viral pneumonia. However, one drawback in all the proposed GAN methods can be a low resolution of generated images ($128^2$ pixel size). Consequently, these synthetically obtained images are difficult to be validated by a radiologist. Generation of high-resolution images is not trivial since the high-resolution images contain detailed features which drastically increase the gradient problems^35^. Due to the large resolution, smaller batches of data need to be used, due to memory bottlenecks, which compromises training stability. As mentioned above, a way to increase the accuracy can also be to focus on binary classification of Covid-19 with two classes of healthy and pneumonia images being labeled as Covid-19 negative. Furthermore, the discriminator of GANs is not powerful enough to exhibit a high classification accuracy since the discriminator architectures of GANs are generally not very deep and, therefore, comparable with other standalone CNN architectures. Hence, further developments of neural network topologies are necessary to combine multi-class classifications with high-resolution images. To the knowledge of the authors, currently available studies in the literature do not address this issue.

This is where the present study comes in. We propose a Residual U-Net GAN based data augmentation to increase the size of the dataset and a novel CNN architecture based Covid-19 detection strategy that is capable to achieve a classification accuracy of over 99%. To account in image classification for the relevant image data, the need for image segmentation for improving the receiver operating characteristic (ROC) score was discussed in^40^. However, in the present study, we use unsegmented images and show, that a reduction of image noise using segmentation is not necessary in our case due to the use of high-resolution synthetic images.

**Figure 1:**
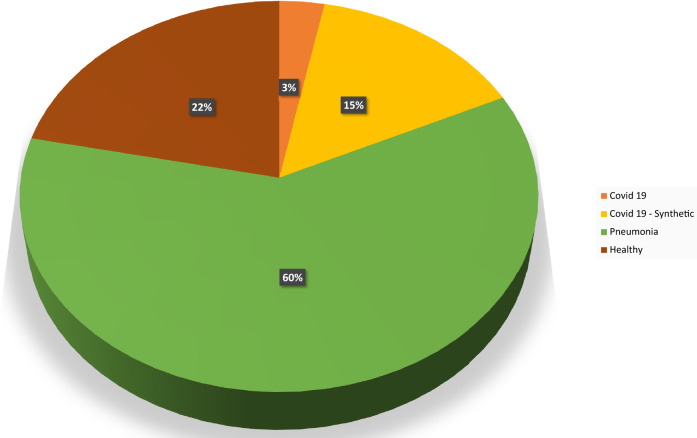
Distribution of data over different classes.

## Dataset

The chest X-ray images used in this study are taken from the public dataset, covidx^24^, compiled of chest X-ray images from Covid-19 affected individuals, healthy individuals and pneumonia-affected individuals. These RGB images with a pixel range of [0, 255] have various resolutions. To train the generative models in this study, all images were converted to grayscale, resized to $1024\times 1024$ pixels, and normalized to have pixel intensities in the [0, 1] range. Since the images have unequal pixels lengthwise and width-wise, to prevent the distortion of images due to resizing from a mxn resolution to hxh resolution, a central crop is employed using a square bounding box. This helps in having an equal number of pixels in length and width wise in the image, thereby preventing distortion when resized to a square resolution. Also, chest X-ray images from Montgomery county dataset and Shenzen dataset^41,42^ have been included to boost the training data. The primary dataset consists of 417 covid affected X-ray images, 8148 pneumonia affected X-ray images, and 2924 healthy X-ray images. Figure 1 shows the distribution of data between different classes obtained from the public datasets. The exact number of images in each class is given in the Table 1

**Table 1:** Categorization of data into classes and sets.

Data distribution		Classes of X-ray images		
		Covid-19	Healthy	Pneumonia
Overall data	Before augmentation	417	2924	8148
Training set	–	2027	2624	7368
Test set	–	390	390	780

## Instance and batch normalized residual U-Net GAN

**Figure 2:**
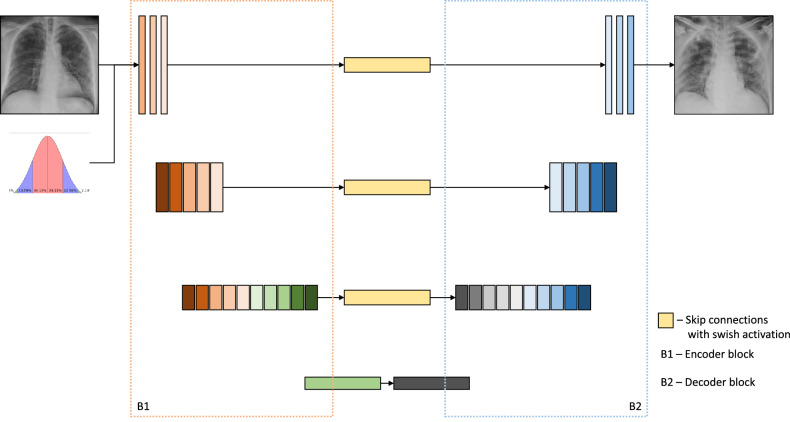
Residual U-Net GAN.

We proposed a modified residual U-Net architecture as the generator, which consisted of an encoder, decoder, and symmetric skip connections between encoder and decoder blocks. The network is instance and batch normalized. The convoluting, conventional CNN blocks of the generator together are called an encoder, and the deconvoluting transpose CNN blocks are called a decoder. The encoder block consists of densely connected convolution blocks that help propagating the input features without any losses. The encoder was trained to compress the key information from the image artifacts to feature representations so that the decoder could regenerate the artifact-free image. The skip connections from encoder to decoder played a key role in reconstructing the fine details of the final image. Each block in the encoder consists of a convolutional layer, an instance normalization layer in the early blocks, batch normalization layer in latter blocks, and a swish activation^43^. The proposed GAN with swish activations and instance normalization can equalize the luminosity of the X-ray images, thereby substantially reducing the randomness caused due to the luminosity and swish activation function has proven to show better performance with deeper networks^43^ as compared to a ReLU activation function. Each block of a decoder consists of a Transposed convolutional layer, a batch normalization layer, a dropout layer for the first three blocks, and a ReLU activation function. Skip connections are provided between all the layers except for the middle. Skip connections help with the vanishing gradients problem and mitigate accuracy saturation or the degradation problem. Each skip connection is provided with a mapping layer called a skip mapper, that performs swish activation. These skip connections provide additional information exchange between the convolution and the deconvolution blocks which results in better convergence. The architecture of proposed IBNRUN GAN is shown in the Fig. 2

The generated image is then passed through a discriminator which differentiates the generated image from the real image. The discriminator is also a GAN, in this case, a PatchGAN. A PatchGAN is a type of discriminator for GANs which classifies the data based on the structure at the local data patches. The patch GANs have proven to be efficient at tasks like object reconstruction from edge maps, photosynthesis, and style transfer implementations like pix2pix. In this study, we build upon the basic U-Net architecture proposed by^44^ incorporating dense connectivity inside the convolution blocks and residual connections to address vanishing gradients and swish activation which tends to work better on deeper models than ReLU^43^ thereby improving the architecture’s efficiency. Each discriminator block consists of a 2D convolutional layer, a batch normalization layer, and a leaky ReLU activation. A Wasserstein loss function is utilized to optimize the parameters of discriminator and generator.

The synthetic images generated by the generator and the real images are fed to the discriminator. The generator then classifies both the real and generated image. The discriminator loss of the real image is called real loss and the generated image is called generated loss. The sum of these two losses constitutes discriminator loss which is used to train and optimize the discriminator. The generator loss function computes the loss as a sum of binary cross-entropy loss of discriminator output and weighted L1 regression of the difference of target and generated images. This generator loss is then used to train and optimize the generator. The generator and the discriminator are trained parallelly and continuously until the generator can produce synthetic images that are indistinguishable from the real images by the discriminator or the reconstruction accuracy is good enough.

1 $$\begin{aligned} \mathop {\mathrm{Min}}\limits _{\mathrm{G}} \mathop {\mathrm{Max}}\limits _{{\mathrm{D}}} V(G,D) = E_x[log(D(x))] +E_z[log(1-D(G(z)))]. \end{aligned}$$

Thus, the learning procedure is a two-player game, where the discriminator and generator compete with each other. The ultimate goal of the training is to make the generator be able to learn to generate a distribution that matches the distribution of the training dataset.

Therefore, the proposed GAN architecture, with instance normalization to equalize luminosity, with batch normalization to prevent overfitting, with densely connected convoluting blocks and residual connections to prevent impeding of information and vanishing gradient problems, and with swish activation that has been to proven better than ReLU in deeper networks, is capable of generating realistic and less random synthetic images.

## Novel CNN based multi-class classification

Convolutional neural networks (CNNs)^45^ have come to be the go-to machine learning approach for the classification and detection tasks. In recent years, deeper networks with narrow kernels and many layers have become popular due to the possibility of having more non-linear activations for the same effective size as of a wider network, thereby improving accuracy^46,47^. Two such widely used state-of-the-art architectures^48^ in the CNNs are ResNet and DenseNet. Though the state of the art networks excel in classification tasks even with hundreds of classes, they have millions of training parameters and consume a lot of memory. Utilizing these models in radiology equipment requires considerably more computation power and time to run the models. Also, the X-ray images used in this study contain a lot of randomness in the luminosity and also in their resolution, since they are compilation of images from different sources and periods of time. The use of Global average pooling^49^ after convolutions, followed by a softmax activation is extremely useful while classifying objects or images of classes with distinct features like cats, dogs, humans, flowers, cars, etc. But in the case of the CXR dataset, the X-rays, although not completely identical, are not trivial like other object detection or classification problems due to the unique and non-trivial nature of the disease markers that are to be identified.

Hence to overcome these above limitations, a novel CNN architecture is proposed with branched convolutions, instance normalization and densely connected layers, respectively. The branched convolutions reduce the overall parameters, thereby keeping the network lighter. To address the problem of randomness of luminosity and brightness in the CXR images, instance normalization is used in the initial layers to equalize the images. Finally, Flatten and dense layers are used to replace the Global average pooling layer to facilitate for fine-tuning of parameters for greater accuracy. Therefore, in this study, a novel, modified version of the state-of-the-art architectures is proposed and applied for the multi-class classification of X-ray images between pneumonia, healthy, and Covid-19 classes.

### CNN architecture

The architecture proposed in this study is based on Densely Connected Convolutional Networks (DenseNet). DenseNets are a class of deep CNNs proposed in^50^. The classical DenseNet is modified to have less training parameters to make it computationally lighter. In a classical DenseNet, each layer connects to every other layer in a feed-forward fashion, meaning that DenseNet has $\hbox {n}(\hbox {n}+1)/2$ connections in total. For each layer, the feature maps of all previous layers are used as inputs, and its feature maps are used as inputs to all subsequent layers, i.e. the $(n+1)$th layer receives the feature maps of all preceding layers as input which modifies $x_{n+1} = H(x_n)$ as

2a $$\begin{aligned} x_{n+1} &= H(x^{'}_{n}). \end{aligned}$$

2b $$\begin{aligned} x^{'}_{n+1} &= {x^{'}_{n}}^\frown x_{n+1}. \end{aligned}$$

where *H* represents $[Normalization \rightarrow Activation \rightarrow Convolution]$ of $x^{'}_{n}$, $^\frown$ represents concatenation of feature maps and $x^{'}_{n}$ $= [x_0^\frown x_1^\frown x_2^\frown \dots ^\frown x_n]$. A branched block of densely connected convolutions is then introduced, which increases the non-linearity of the network while lowering the total training parameters. The branched convolution blocks are incorporated into the network in the second and third blocks to just serve the purpose of making the model lighter without compromising accuracy. This modifies Eq. (2) for branched convolution block as

3a $$\begin{aligned} {(x_{n+1})}_i &= H_i(x^{'}_{n}) \qquad {\forall i \in [1,4]} \end{aligned}$$

3b $$\begin{aligned} x_{n+1} &= [H_1^\frown H_2^\frown H_3^\frown H_4] (x^{'}_{n})\end{aligned}$$

3c $$\begin{aligned} x^{'}_{n+1} &= {x^{'}_{n}}^\frown x_{n+1}. \end{aligned}$$

**Figure 3:**
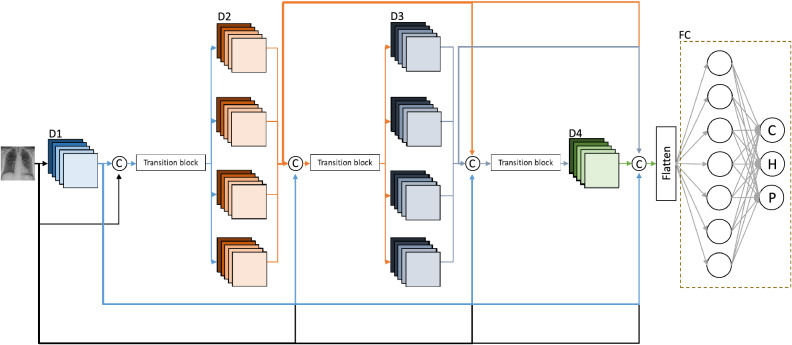
Block diagram of the proposed architecture.

**Figure 4:**
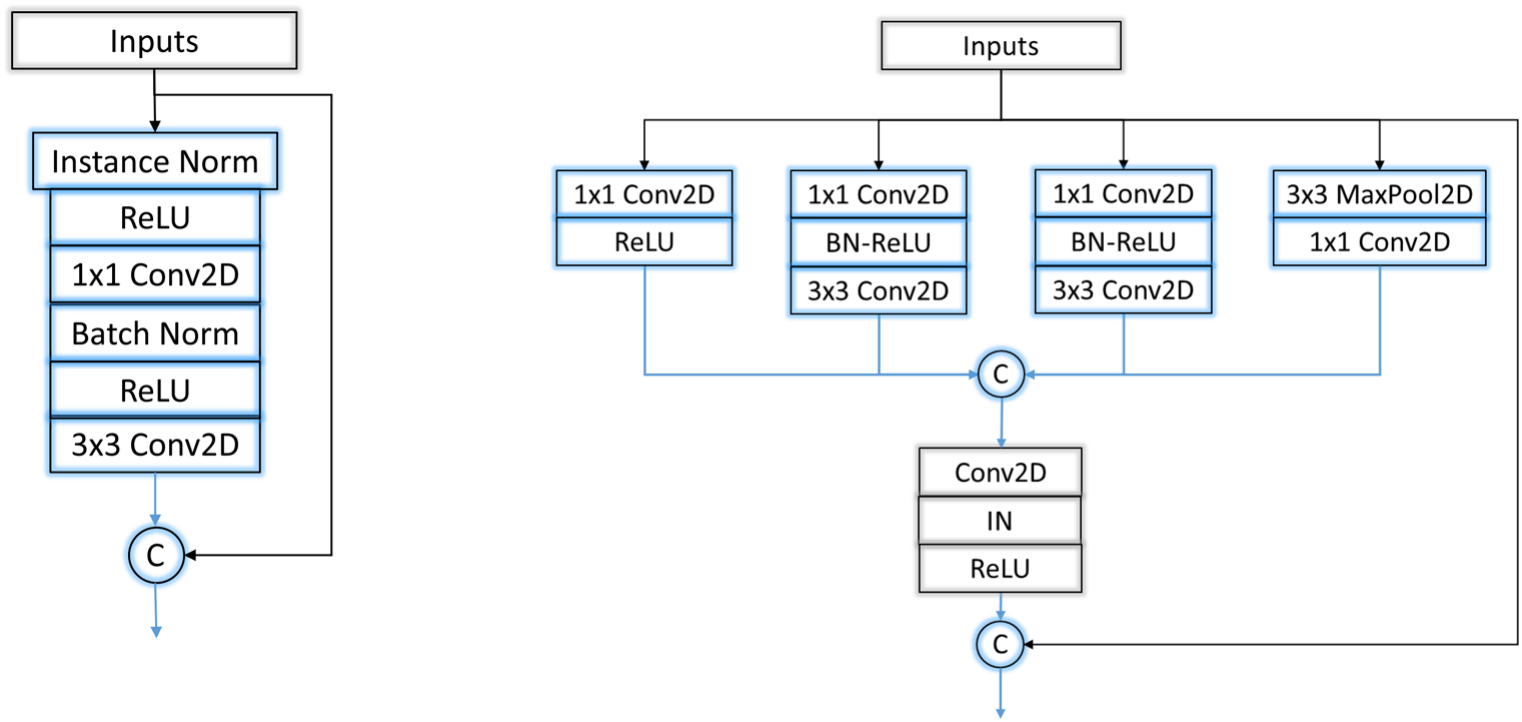
State of the art DenseNet architecture (left) and architecture of convolution blocks of proposed architecture (right).

Classical convolution blocks make up the first dense block with instance normalization^51^ instead of batch normalization and also, in the last dense block to have more training parameters and higher information propagation to the final layers. The proposed architecture with dense branched convolutions is shown in Fig. 3. Here D1, D2, D3 and D4 represent the dense blocks, where D2 and D3 represent the branched convolution blocks. The architecture of the branched convolution blocks is shown in Fig. 4.

## Data pre-processing and experiment methodology

**Figure 5:**
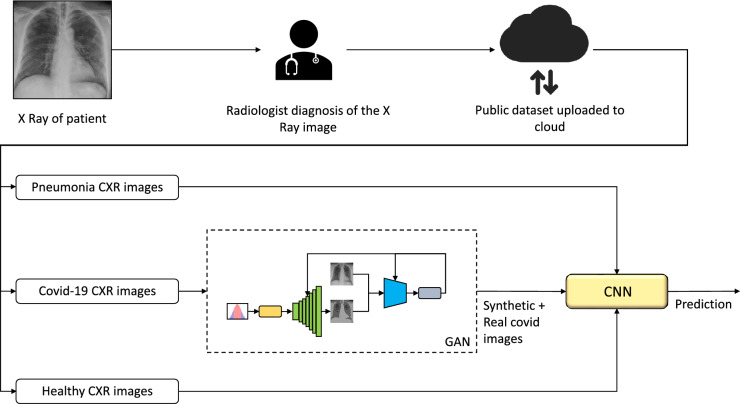
Workflow

The workflow is shown in the Fig. 5. All methods were performed in accordance with the relevant guidelines and regulations. We start with collecting primary data from three public sources such as Covidx dataset^24^, Shenzen and Montgomery county datasets^41,42^. The primary dataset contains 417 Covid-19 affected X-ray images, 8148 pneumonia affected X-ray images, and 2924 healthy X-ray images. Since CNNs requires a lot of data to train and achieve good classification accuracy, the proposed IBNRUN GAN is developed to generate synthetic Covid affected X-ray images to increase the training dataset and get better accuracy for classification. The GAN is modelled using Tensorflow 2.8 and Keras libraries. Mixed precision layers are employed to minimize memory consumption wherever needed. Consequently, 2000 synthetic Covid X-ray images are generated. The class of Covid consists of 2027 images that are used for training, out of which 2000 are synthetically generated and 27 are from the Covidx dataset. All the other real Covid X-ray images are used for evaluating the model. To achieve an optimised hit rate, a nearly proportional number of healthy and Covid-infected images was used to balance the data set. As mentioned in the Dataset section, all the images are cropped to a square bounding box with a side length equal to the size of the smaller of lengthwise or widthwise pixels to prevent distortion. Then the images are resized to $224 \times 224$ pixels using a convolution-based high-quality Lanczos filter to preserve quality after downscaling. The images are then normalized to [0,1]. These three classes of images are then used to train the two proposed very deep CNN models. A test set of 390 Covid X-ray images, 390 healthy X-ray images, and 780 pneumonia X-ray images are used for evaluation. All the networks used in this study are coded using Tensorflow 2.7 and Keras libraries. The models are trained using an Nvidia RTX 3090 GPU with 24GB of memory.

## Results

**Figure 6:**
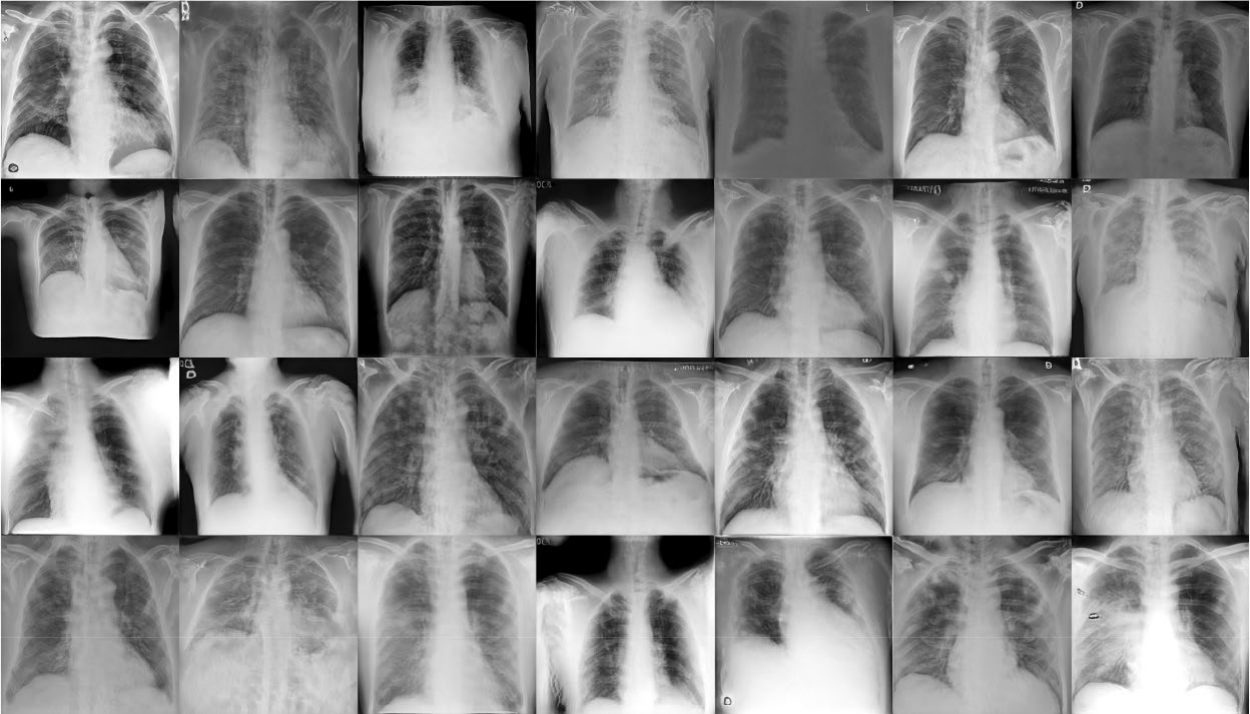
Synthetic images generated by GAN.

**Figure 7:**
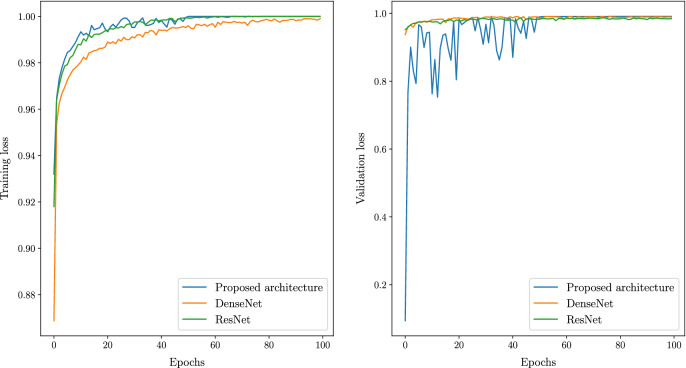
Comparison of accuracy vesus epochs between proposed and state of the art architectures.

**Figure 8:**
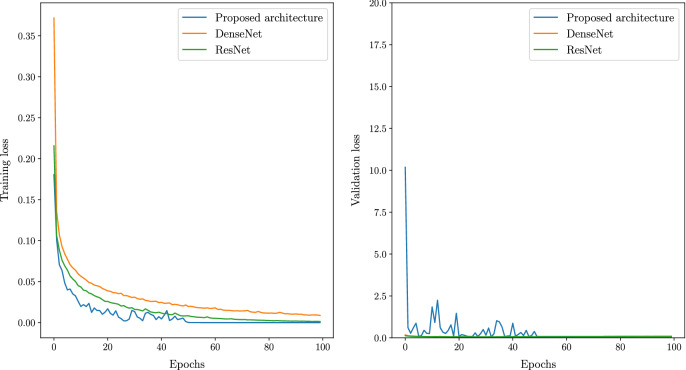
Comparison of loss versus epochs between proposed and state of the art architectures.

The proposed IBNRUN GAN is trained using the 417 Covid-19 affected chest X-ray images that have been taken from the public dataset. The GAN is trained for 100k epochs and CNNs are trained for 200 epochs respectively. 2200 synthetic Covid-19 images are generated from the trained GAN and 2000 images are selected from this pool of 2200 images. 27 real X-ray images are then added to the Covid-19 training pool of CNN along with the synthetic images. The rest of the Covid-19 X-ray images are used for evaluating the trained models. A sample of the generated synthetic images is shown in Fig. 6. The accuracy and loss plots of the proposed architecture along with their state of the art counterparts are shown in Figs. 7 and 8.

**Figure 9:**
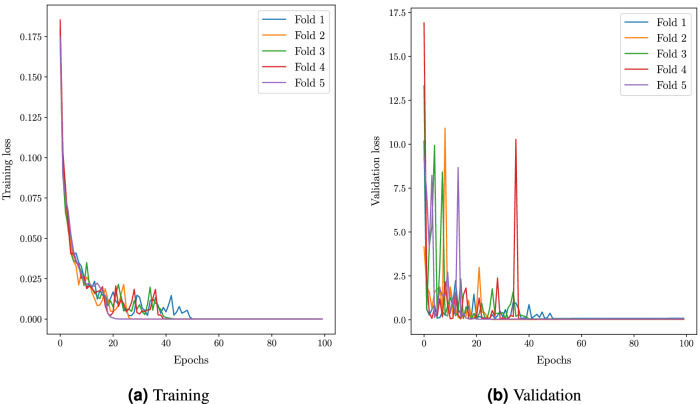
5 Fold cross valdiation of proposed architecture.

Even though batch normalization and dropout layers are incorporated to prevent overfitting, a 5-Fold cross-validation is performed to study the sensitivity of the model to a different selection of training and validation sets. The loss and accuracy plots from the sensitivity analysis are shown in Fig. 9 respectively. All the models are then evaluated using a test set of 390 covid images, 390 healthy images and 780 pneumonia images respectively.

**Figure 10:**
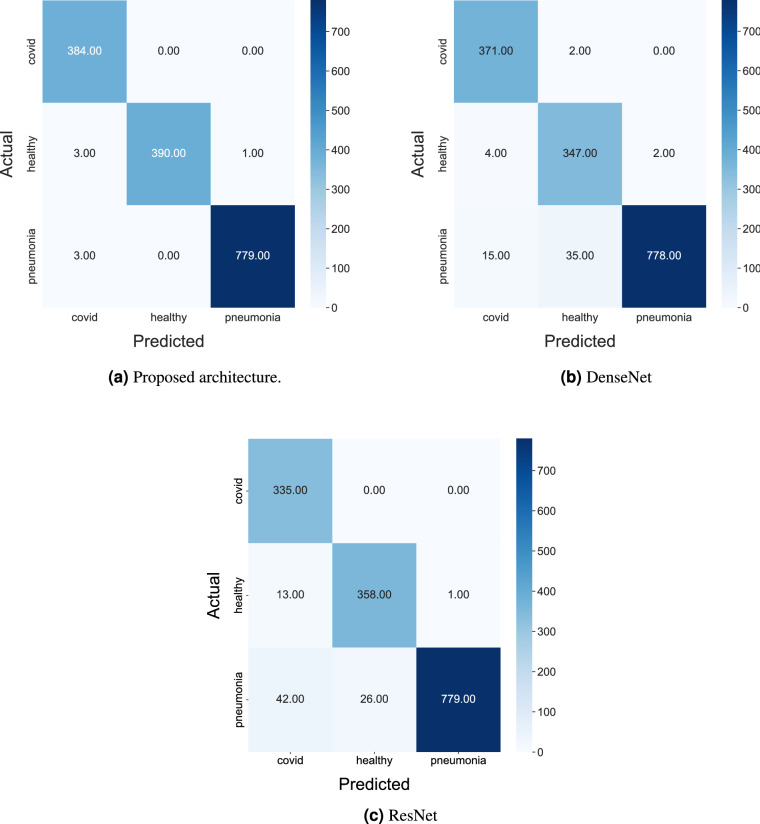
Confusion matrices of all the architecture understudy showing metrics by number of test samples.

**Figure 11:**
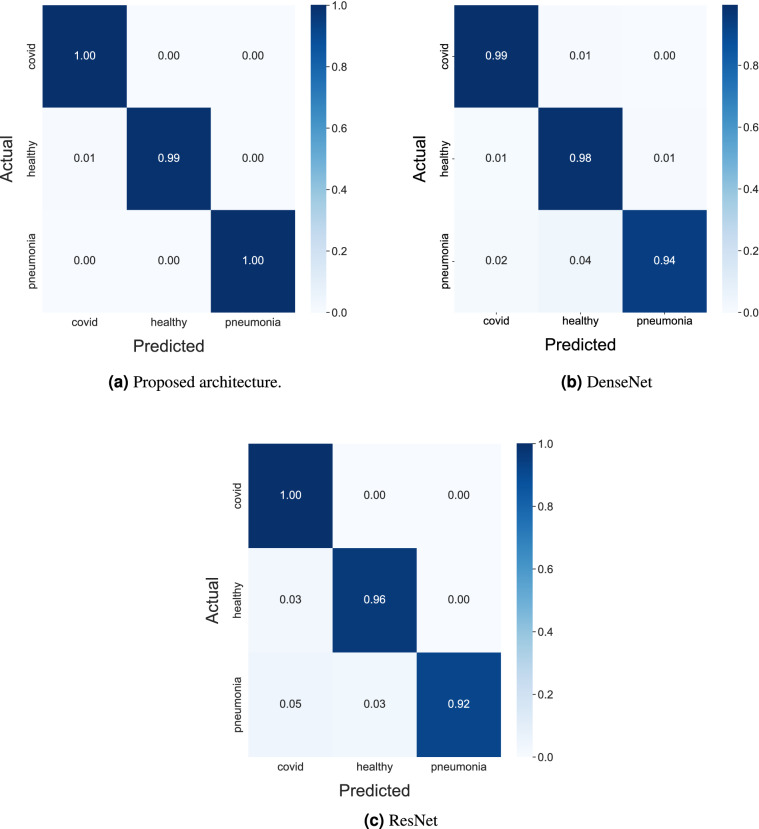
Confusion matrices of all the architecture understudy showing metrics by percentage.

In Figs. 10 and 11, confusion matrices are shown to check the true positives and false positives of the models. It can be observed from Figs. 10a and 11a that the number and percentage of false positive and false negative prediction rate of the proposed CNN architectures on the test set is extremely close to zero. The model perform the classification very efficiently with an accuracy of 99.28%. Whereas for the other models, it can be observed that the performance is well below that of the proposed architecture from Figs. 10b,c and 11b,c.

The true positives (*tp*), true negatives (*tn*), false positives (*fp*) and false negatives (*fn*) are obtained from the confusion matrix. Since this is a multi-class classification, one versus all approach is used where, in the first case metrics for Covid-19 versus. all are computed, healthy versus all in second and pneumonia versus all in the third respectively. From these metrics, performance metrics such as Precision, Recall, Specificity and F-Score are calculated as follows.

4a $$\begin{aligned} Precision~(P)&= \frac{tp}{tp+fp}. \end{aligned}$$

4b $$\begin{aligned} Recall~(R)&= \frac{tp}{tp+fn}.\end{aligned}$$

4c $$\begin{aligned} Specificity~(S)&= \frac{tn}{tn+fp}.\end{aligned}$$

4d $$F{\text{-}}Score = \frac{{2*P*R}}{{P + R}}.$$

The metrics for each of the three classes are calculated and the average of these metrics are taken to represent the overall performance of the model. The classification performance of the proposed and state of the art models are given in Table 2.

**Table 2:** Performance metrics of the proposed and state of the art models.

Architecture		Performance metrics (%)				
		Accuracy	Precision	Recall	Specificity	F-Score
Proposed architecture	Fold 1	98.59	98.33	98.68	99.30	98.50
	Fold 2	99.49	99.36	99.45	99.76	99.40
	Fold 3	99.42	99.27	99.41	99.73	99.34
	Fold 4	99.23	99.06	99.20	99.63	99.12
	Fold 5	99.55	99.44	99.53	99.79	99.49
DenseNet	–	93.21	91.03	95.45	97.54	93.18
ResNet	–	92.24	89.70	94.60	98.11	92.08
Karaci et al.^25^	–	93.46	91.28	95.52	97.14	93.00
Ozturk et al.^26^	–	93.20	91.06	95.66	97.01	92.58
Ioannis D. et al.^52^	–	93.14	90.94	95.13	96.68	92.62
Sarki et al.^53^	–	92.75	90.72	95.03	96.73	92.48

**Figure 12:**
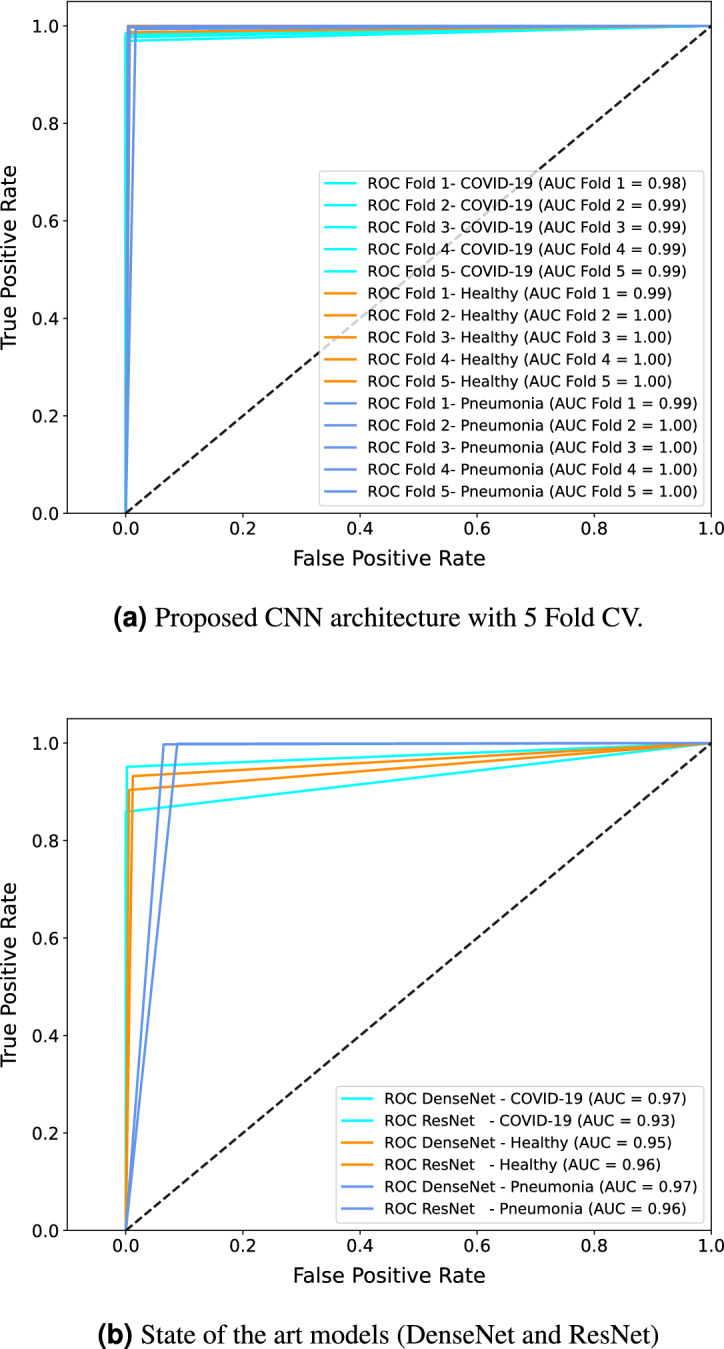
ROC curves and AUC of all the models under study.

Apart from the state-of-the-art classical CNN models, state-of-the-art classification models used for disease diagnosis of Covid have also been compared. The models were reconstructed in Tensorflow based on the architecture described in the respective articles and have been trained on the same dataset used in the study with images augmented using IBNRUN GAN. The performance metrics of the state-of-the-art COVID classification models have been shown in Table 2. The relation between sensitivity (fraction of true positives) and specificity (fraction of true negatives) of the trained models on the test set is assessed graphically by Receiver operating characteristic (ROC) curves. One versus all approach has been used to plot the ROC curves. The usefulness of the model is given by the area under the ROC curve (AUC) with 1.0 being the best measure. The plots in the Fig. 12 showcase ROC, using one versus rest approach with Covid versus rest, healthy versus rest, and pneumonia versus rest. For proposed architecture, ROC plots are made during the 5-Fold cross-validation to assess the model’s sensitivity to new data. It can be observed from the Fig. 12a,b that the proposed models perform much better than the state of the art models while having almost 40% less parameters.

## Discussion

In the current study, the IBNRUN GAN architecture effectively produced synthetic images that boosted the training set and had a realistic appearance. The network has been designed to deal with the randomness of X-ray images. Here, using the proposed CNN architectures, a multiclass classification is carried out using three classes: Covid, healthy, and pneumonia. These are then contrasted with state-of-the-art models. The proposed novel architecture includes instance normalization, branched convolution blocks, and dense layers to make the models lighter and more accurate. In comparison to the most advanced versions, this led to models that were 40% lighter. The proposed model has an execution rate of 25ms/step where as the state-of-the-art DenseNet architecture has an execution rate of 47ms/step. This shows that the proposed model is faster in execution as compared to the state-of-the-art models. That said, there has not been a gain in training speed due to the use of instance normalization since it is computationally expensive as compared to batch normalization during training. Nevertheless, both the proposed model and the state-of-the-art counterpart consumed 63sec/epoch while training. In^40^, the significance of segmentation is discussed. Although lung segmentation is not done, it can be shown that the network still gets strong ROC and AUC values as shown in Fig. 12. Furthermore, because of the limited Covid X-ray image dataset that is available for training, we outperform a standalone CNN network in terms of test accuracy. The proposed method has the best testing accuracy (99.2%) in comparison to the literature due to its novel IBNRUN GAN with instance normalization and swish activations and CNN architectures. The authors are aware that the current study is based on a closed data set, nevertheless. Additionally, it is noted that the human body is superimposed onto a 2D image, which causes the X-ray images to have a great deal of unpredictability. In order to investigate the proposed method close to application, a medical study using X-ray images from routine clinical practice would be ideal. However, the current study developed the scientific foundation which can be used in clinical studies.

## Conclusion

A novel artificial neural network architecture was suggested in the current study to identify Covid-19 infection in the lungs using X-ray pictures. The developed technique is suggested as a rapid diagnostic tool due to the high hit rate of 99% of the fully trained network model. Additionally, it was possible to differentiate between Covid, pneumonia, and healthy lungs. However, the level of information in the photos may be what distinguishes pneumonia from Covid infections. Consequently, a significant number of data was required for a successful categorization between these three categories of lung imaging. For this reason, synthetic X-ray images were produced using a Residual U-Net based GAN model. Following this methodology, a very deep CNN model with high accuracy was suggested. It was trained using both available real images from public data banks and generated images from GANs. radiology equipment and the trained CNN model can be used together to quickly diagnose X-ray images as soon as they are taken. The proposed models have the better accuracy as compared to state of the art models while having 40% less parameters.

## Data Availability

The datasets used and analysed during the current study available from the covidx^24^ dataset complied of chest X-ray images with Covid-19 infections, healthy and pneumonia which can be accessed at https://github.com/ieee8023/covid-chestxray-dataset. Also, chest X-ray images from Montgomery county dataset and Shenzen dataset^41,42^ have also been utilized which can be downloaded from https://www.kaggle.com/datasets/kmader/pulmonary-chest-xray-abnormalities. The sources are publicly licensed and available for download from the website of National Institute of Health, Maryland, USA.
